# Integration of Genome and Chromatin Structure with Gene Expression Profiles To Predict c-*MYC* Recognition Site Binding and Function

**DOI:** 10.1371/journal.pcbi.0030063

**Published:** 2007-04-06

**Authors:** Yili Chen, Thomas W Blackwell, Ji Chen, Jing Gao, Angel W Lee, David J States

**Affiliations:** 1 Bioinformatics Program, University of Michigan Medical School, Ann Arbor, Michigan, United States of America; 2 Department of Human Genetics, University of Michigan Medical School, Ann Arbor, Michigan, United States of America; 3 Pharmacology Department, University of Michigan Medical School, Ann Arbor, Michigan, United States of America; University of California San Diego, United States of America

## Abstract

The MYC genes encode nuclear sequence specific–binding DNA-binding proteins that are pleiotropic regulators of cellular function, and the c-*MYC* proto-oncogene is deregulated and/or mutated in most human cancers. Experimental studies of MYC binding to the genome are not fully consistent. While many c-*MYC* recognition sites can be identified in c-*MYC* responsive genes, other motif matches—even experimentally confirmed sites—are associated with genes showing no c-*MYC* response. We have developed a computational model that integrates multiple sources of evidence to predict which genes will bind and be regulated by MYC in vivo. First, a Bayesian network classifier is used to predict those c-*MYC* recognition sites that are most likely to exhibit high-occupancy binding in chromatin immunoprecipitation studies. This classifier incorporates genomic sequence, experimentally determined genomic chromatin acetylation islands, and predicted methylation status from a computational model estimating the likelihood of genomic DNA methylation. We find that the predictions from this classifier are also applicable to other transcription factors, such as cAMP-response element-binding protein, whose binding sites are sensitive to DNA methylation. Second, the MYC binding probability is combined with the gene expression profile data from nine independent microarray datasets in multiple tissues. Finally, we may consider gene function annotations in Gene Ontology to predict the c-*MYC* targets. We assess the performance of our prediction results by comparing them with the c-*myc* targets identified in the biomedical literature. In total, we predict 460 likely c-*MYC* target genes in the human genome, of which 67 have been reported to be both bound and regulated by MYC, 68 are bound by MYC, and another 80 are MYC-regulated. The approach thus successfully identifies many known c-*MYC* targets and suggests many novel sites. Our findings suggest that to identify c-*MYC* genomic targets, integration of different data sources helps to improve the accuracy.

## Author Summary

c-*MYC* is an important proto-oncogene that controls the expression of many other genes, and MYC regulation is deranged in many cancers. Identifying c-*MYC* target genes is one of the key steps to understand both the biological role and molecular mechanism of c-*MYC* action. Defining the complete list of c-*MYC* target genes and categorizing them as genes that are directly and indirectly modulated remains a challenge. Computational models also help us to understand the mechanisms modulating c-*MYC* function. We describe a method to predict where MYC will bind in the genome and which c-*MYC* binding sites will be biologically active. The method integrates multiple sources of data, including both genome sequence and functional annotations, to predict that 460 genes are direct c-*MYC* targets. These include many genes previously known to be c-*MYC* targets as well as 245 novel direct c-*MYC* targets. Using multiple, independent gene-expression datasets improves the sensitivity and specificity of the prediction and demonstrates significant tissue-specific variation in c-*MYC* action at different genes. Our study suggests that chromatin state plays an important role in modulating both c-*MYC* binding-site activity and the functional consequences of c-*MYC* binding.

## Introduction

MYC plays a critical role in regulating cell proliferation, growth, apoptosis, and differentiation. Human malignancies are often associated with aberration of the c-*MYC* gene [1–3]. The diversity of its functions has been attributed to c-*MYC*'s ability to activate or repress the transcription of an extensive array of target genes mediating a wide range of cellular activities [4–6]. c-*MYC'*s actions are mediated by sequence-specific binding of the c*-MYC* protein, dimerized with its partner MAX, to DNA elements called E-boxes with the core sequence motif 5′-CACGTG-3′ [7–9]. Binding of the MYC–MAX heterodimer to a target gene can directly activate or repress transcription, but many E-boxes do not bind MYC, and in many experimentally confirmed cases, MYC binding is not associated with changes in gene expression. Identifying functional MYC binding sites and target genes is a critical step in understanding both the biological role and molecular mechanism of MYC action.

mRNA expression studies have identified many target genes activated or repressed by c-*MYC* in various animal and human cells or cell lines. The number of experimentally validated c-*myc* targets are expanding rapidly thanks to the use of high throughput methods [10–13]. The recent studies of Basso et al. [14] and Remondini et al. [15] suggest that the potential list of c-*MYC* targets could be much larger than what was previously anticipated. However, different experimental and theoretical studies give quite different findings, and much work remains to be done to define the complete set of c-*MYC* targets.

Gene expression studies alone cannot discriminate between direct and indirect targets of c*-MYC* action, although network-based inference of direct action has been proposed [14]. Recently, chromatin immunoprecipitation (ChIP) and whole genome scale analysis of methylation status have emerged as new sources of relevant data for the analysis of genomic regulatory elements.

Theoretical analysis complements and extends experimental study, and several researchers have attempted to predict c*-MYC* target genes using computational methods. By searching human transcript sequence, analyzing E-box location, and using genomic sequence evolutionary conservation, Schuldiner et al. [16] identified 12 putative targets, two of which were confirmed by subsequent experimental analysis. Zeller et al. [17] built a database of c-*Myc* responsive genes that have been reported in publications and supported by multiple lines of evidence. They then identified seven out of 12 candidate genes in this database using phylogenetic analysis, and they confirmed six of these predictions using ChIP. The use of evolutionary conservation is also supported by Haggerty [18] who categorized c-*MYC* targets into two classes: Class I, to which the majority of genes belong, has E-boxes that are evolutionarily conserved; and Class II, which includes genes with no region of homology at or flanking the genomic regions that exhibit MYC binding.

The promoter regions of c-*MYC*–regulated genes often contain E-box sequences that bind MYC with high occupancy. Li et al. [17] analyzed genomic binding sites for c-*MYC* in Burkitt lymphoma cells and found a strong correlation between MYC DNA binding and gene transcription, strengthening the view that high binding occupancy of a c-*MYC* site near a gene's promoter region is, to some extent, a sign of a c-*MYC* target gene. However, this is not a hard and fast rule, and many sites that bind MYC with high occupancy are not associated with c-*MYC* target genes. Fernandez et al. [19] performed a large-scale assay for genomic Myc binding sites in vivo by quantitative ChIP. They found that promoter E-boxes are distributed in two groups differing in MYC binding occupancy. The strongest DNA-sequence characteristic of high-affinity/high-occupancy targets was location of the E-box within a CpG island. This observation can be partially explained by the fact that most CpG dinucleotides in the mammalian genome are subject to cytosine methylation [20,21], but the methylation of the CpG dinucleotide in the consensus myc binding site sequence reduces the binding affinity of myc–max dimers for the target DNA [22,23]. Further, DNA methylation is often coupled to and associated with histone methylation and the formation of heterochromatin [24]. Recent work by Ernesto [25] shows that target sites are only recognized MYC by whether they are packaged in chromatin bearing high H3 K4/K79 methylation and H3 acetylation. This is true for both classic E-box (CACGTG) and alternative sequence sites.

With the abundant volumes of experimental data on c-*MYC* target gene expression and in vivo binding of MYC to the genome, there is an increasing need to integrate these information resources and to classify c-*MYC* responsive genes into direct and indirect targets. In this paper, we identified c-*MYC* target genes using a computational approach that draws on data from multiple sources including gene expression profiling, gene annotations, ChIP, sequence conservation, and sequence composition. First, we developed a computational model to predict the likelihood of CpG methylation. Next, we developed a computational strategy to predict which E-box sites were likely to be functional MYC binding sites. Finally, the binding predictions were integrated with gene expression and gene annotation data to identify direct and indirect c-*MYC* target genes. The performance of these tools was validated by comparison with multiple experimental datasets. Our method is able to successfully predict the occupancy of the binding sites as revealed by CHIP. Although this computational method was specifically built on c-*MYC* data, it also provides useful information on the binding of cAMP-response element-binding protein (CREB) [26], another transcription factor whose binding is sensitive to DNA methylation. After further integration with gene expression data from different tissues and datasets, and the Gene Ontology (GO) annotations for c-*MYC* targets, we identified 460 likely c-*MYC* target genes. Of these genes, 215 have been previously identified as MYC bound or regulated and 245 are novel. Our study shows that integrating multiple data sources improved the prediction specificity of MYC binding site prediction. Similarly, using gene expression profiles from several independent studies improved the sensitivity for target gene prediction. Our analysis suggests that much of the variation between microarray-based gene expression assays may be due to limitations of the technology. In addition, there appears to be a significant tissue-specific component to the responses of some c-*MYC* target genes.

## Results

### Computational Prediction of Genome CpG Islands and Hypomethylation Regions

The performance of the fifth-order Markov model for genomic methylation status was tested using an independent dataset from the Human Epigenome Project [27]. We found that 86.4% of these unmethylated sites fell within our predicted hypomethylation regions. In contrast, only 22% of the hypermethylated CpG sites were within the predicted hypomethylated regions (*p* < 2.2e−16). We applied this model to predict the CpG islands and hypomethylation regions on the human genome. On the human repeat masked genome NCBI35, we found that 0.71% of the human genome sequences were CpG islands and 1.07% of the genome sequences were predicted to be hypomethylated regions, with 82% of the CpG islands falling within hypomethylated regions. As anticipated, promoter regions had much higher percentages of CpG islands and hypomethylated regions than the whole genome. Based on our prediction results, 43% of the human genes had CpG islands and 46% of the genes had hypomethylated regions within 5 Kb upstream of their transcription start sites. In the 5 Kb upstream of the transcription start sites, 4.4% of the sequence was covered by a CpG islands and 6.7% of sequences were predicted to be hypomethylated.

### Computational Prediction of High Occupancy MYC Binding Sites

In this step we identified sites in the human genome where MYC is expected to bind in vivo with high occupancy. Candidate MYC binding sites were identified by scanning the complete human genome sequencing using the TRANSFAC MATCH algorithm [28] and the MYC–MAX position specific weight matrix (PSWM) MA0059 [29] from the JASPAR database [30,31]. Sites that achieved a matrix score of 0.8 are referred to as motif matches. Four additional sources of data were used to define a subset of these motif matches that are likely to bind MYC in vivo: proximity to transcription start sites, proximity to CpG islands, predicted hypomethylation, and evolutionary conservation.

To train our algorithm, we began with the Fernandez et al. [19] data for genomic Myc binding sites in live human cells. This study examined MYC binding to more than 700 E-box sequences in vivo by quantitative ChIP. The authors found that promoter E-boxes were distributed in two groups that bound MYC at distinct frequencies. In the Fernandez et al. dataset, we only used sites where the PCR primers and the associated E-box could be mapped back to the human genome as an exact sequence match. Further, sites were excluded when the dataset contained multiple contradictory assay results. This filtering process resulted in a set of 493 binding sites where ChIP was measured in either U937 or HL60 cell lines (for 40% of the sites, data is available and consistent across both cell lines). A high quality training dataset was defined using sites where ChIP results were consistent across more than one assay. The training set had 43 nonredundant high-occupancy sites and 90 nonredundant low-occupancy sites.

**Figure 1:**
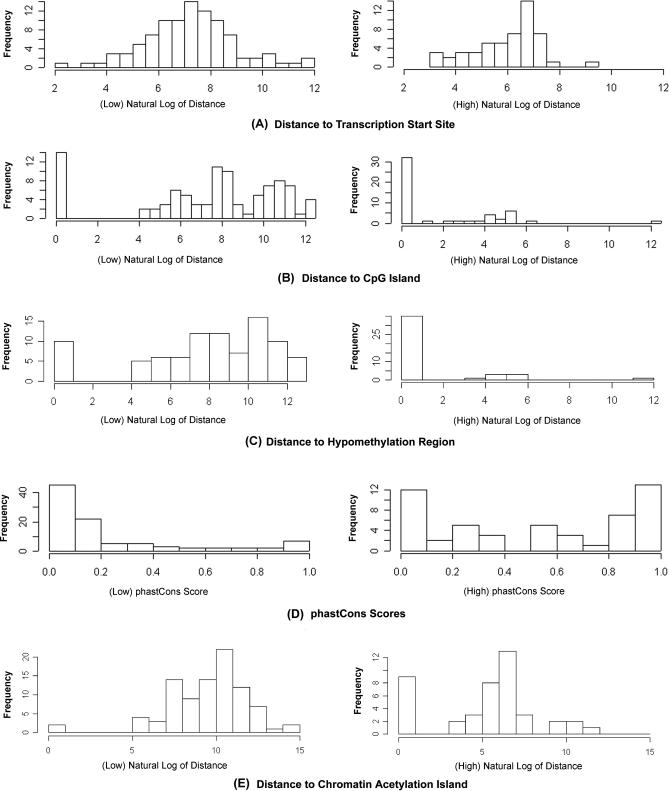
Distribution of Sequence Attributes Distribution of sequence attributes used as input for the Bayesian classification. (A) Distribution of distances to transcription start site. (B) Distribution of distances to nearest CpG islands. (C) Distribution of distances to nearest hypomethylation region. (D) Distribution of PhastCons scores from the UCSC genome database. (E) Distribution of distances to nearest chromatin acetylation island. Sequence distance is shown in base pairs. All of the distances were natural log transformed.

Using a Bayesian network classifier, we integrated this high quality training subset of the ChIP dataset with our hypomethylation analysis and genomic sequence conservation data. The resulting classifier predicts which sites in the genome are likely binding MYC with high occupancy. In the training process, we looked for the most important factors determining MYC binding to DNA. The Kolmogorov–Smirnov test showed that the following factors differed significantly (*p* < 0.01) between the high- and low-occupancy sites: distance to transcription start site (Figure 1A), distance to nearest CpG island (Figure 1B), distance to nearest hypomethylation region (Figure 1C), phastCons scores [32] (Figure 1D), and distance to nearest chromatin acetylation island (Figure 1E). The CpG islands and hypomethylation regions near or overlapping with the above MYC binding sites were predicted with our Fast Motif Analyzer (FMA), described in the Materials and Methods section. The acetylation island information was derived from the Roh et al. high-resolution genome-wide mapping of Lys 9 and Lys 14 diacetyl histone H3 in resting and activated human T cells [33]. PhastCons scores identify evolutionarily conserved elements using a multiple alignment of genomic sequence weighted using a phylogenetic tree. The PhastCons score is a base-by-base conservation score that can be interpreted as the probability that each base is in a conserved sequence element [32]. This score is chosen as a measurement of sequence conservation over other alternatives because it is available through the widely used University of California Santa Cruz Genome Browser, and could be readily incorporated into our model.

**Figure 2:**
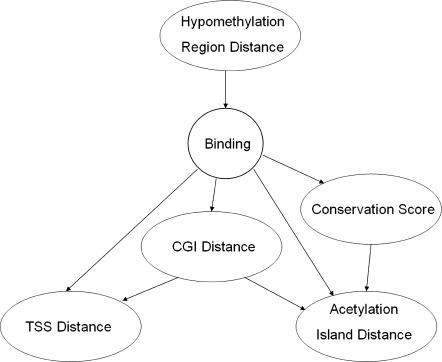
Bayesian Network Classifier Topology of the Bayesian network classifier for high-occupancy MYC binding obtained by training. The detailed parameters for the model are provided in Figure S2.

Using these datasets and a supervised classification approach [34], we evaluated several algorithms for the prediction of high-occupancy MYC binding sites. The best classification results were obtained using a Bayesian network classifier (see Materials and Methods). This method assigned a probability for every site in the genome. We predicted a site to have high binding if this probability was above 0.5. The Bayesian network shown in Figure 2 gave the best results on the high-quality training dataset. A 10-fold cross-validation showed a precision of 0.88 and a recall of 0.98 on the high-occupancy binding sites prediction. When we applied this classifier to Fernandez's entire dataset, it correctly identified 130 of the 183 high-occupancy sites and 258 of the 310 low-occupancy sites.

To further verify that our algorithm was able to predict high-occupancy MYC binding sites, we evaluated performance of independently obtained test data. We considered binding sites within ±3 Kb of transcription start sites, because almost all of the sites in the training data are within this region. We used the MYC–MAX matrix MA0059 [29] from the JASPAR database [30] to identify putative MYC binding sites, and we applied the rules described above to sort these sites into groups predicted to bind MYC with high and low occupancy. Using TRANSFAC MATCH to analyze the complete human genome sequence, we identified 89,560 MYC sites within ±3 Kb of a transcription start site for 14,387 genes. From these candidate sites, our method classified 14,638 sites in 5,276 genes as likely to bind MYC with high occupancy.

We assessed the reliability of these predictions by comparing them with two independently published experimental datasets. One dataset is from Zeller et al. [35] using ChIP–PET to map genomic c-*MYC* binding sites in human B cells. This study the identified loci as PET sequence tag clusters with varying numbers of tags per cluster. More tags matching a cluster increases the reliability of binding site identification. Zeller's paper defined 964 PET-2+ clusters with two or more tags per cluster falling within 3 kb of a TSS. PET-2+ may contain a significant number of false positive identifications. Zeller et al. also defined 113 PET-3+ cluster with three or more tags per cluster and within 3 kb of a TSS. These are believed to be highly reliable identifications. For the second evaluation dataset, we used high-density oligonucleotide array ChIP–chip data from Cawley et al. [36]. Looking only at Chromosomes 21 and 22, Cawley et al. defined 181 high-occupancy MYC binding segments within 3 kb of a TSS.

In the experimental datasets, MYC binding is localized to a segment of genome, but not necessarily a single E-box. We refer to these segments as “MYC binding loci” and compare these with our predictions at the gene level. Most of the experimentally defined MYC binding loci are associated with a single gene. Table 1 compares the experimentally defined MYC binding loci that are within 3 Kb of a transcription start site with those that were predicted by our methods. First, it is apparent that the different experimental assays yield markedly different results. Only one of the 113 Zeller PET-3+ loci was also scored as a high-occupancy binding site by Fernandez et al. Similarly, only one of six Zeller PET-3+ loci on Chromosomes 21 and 22 was identified as a high-occupancy site by Cawley et al., and only four of the 181 binding loci identified by Cawley et al. were scored as high occupancy in the Fernandez dataset. Comparing our predictions with the experimental datasets, we see higher levels of agreement than the experimental datasets show among themselves. This is in part a consequence of the fact that our method predicted a larger number of loci (5,276) than are observed in the experimental datasets. Interestingly, the fractional overlap between our predictions and the Zeller et al. dataset increases as we consider more confident clusters. Whereas only about a quarter of the PET-2+ clusters contain a predicted high-occupancy site, half of the PET-3+ cluster and all of the PET-4+ clusters do. One-third of the loci identified by Cawley et al. on Chromosomes 21 and 22 contain a site predicted to bind MYC with high occupancy, and more than a quarter of our predicted high-occupancy sites on these chromosomes are confirmed by Cawley et al.

**Table 1:**
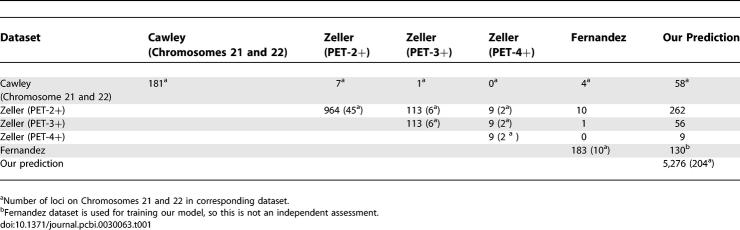
Comparison of Predicted MYC Binding Loci with Published Experimental Datasets

To extend the analysis to more tissues and cell types, we used data from the MYC Target Gene Database. We compared predictions for genes annotated as c-*MYC* targets with predictions for a collection of genes selected at random from the human genome. The MYC Target Gene Database [17] includes more than 1,000 putative MYC target genes reported to be either regulated or bound by Myc. These MYC target genes tend to have more MYC recognition sites than do randomly selected genes. Table 2 shows the performance of our algorithm in the prediction of high-occupancy binding sites. Each gene in Table 2 contains at least one putative MYC site predicted by a motif match. The MYC target genes are compared with a set of 1,000 randomly selected genes containing 6,259 MYC sites, which served as the control group. Second, we chose 589 genes showing myc binding from the MYC Target Gene Database that were not in Fernandez's dataset. Third, we chose 417 genes showing Myc regulation for which no binding data was available. The fourth set consisted of 98 genes in MYC Target Gene Database showing both myc binding and myc regulation that were not in Fernandez's dataset. For each group of genes, we used our algorithm to predict the occupancy of each MYC recognition site. Statistical significance was assessed using Fisher's exact test on each test group against the control group. We found that each of the putative c-*MYC* target groups had a significantly higher percentage of high-occupancy binding sites than the genes in the randomly selected group. This demonstrates that our algorithm is able to discriminate biologically functional high-occupancy sites from low-occupancy, presumably nonfunctional binding-site motif matches.

**Table 2:**
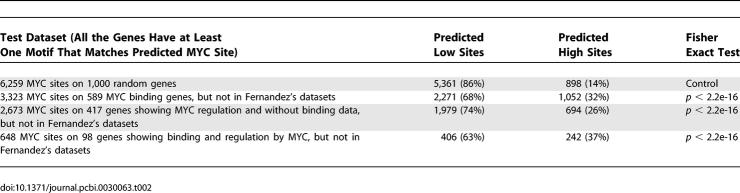
Performance on MYC High-Occupancy Sites Prediction

We next asked what genes were associated with the high-occupancy binding sites. Many genes have multiple MYC binding sites, but little information is available on how multiple MYC sites affect each other's binding. Therefore, in our study we treated all the genes having high-occupancy binding sites as potential MYC binding genes. Table 3 shows the result of these predictions. The results show that known c-*MYC* targets were predicted to bind MYC with a significantly higher frequency than random genes do. Thus, our method has significantly higher accuracy in discriminating the MYC binding genes than does motif match alone.

**Table 3:**
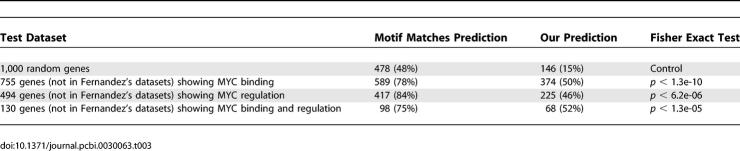
Performance on MYC Binding Gene Predictions

Although our method was built using c-*MYC* data, the attributes used in the model are general, so we anticipated that the method might also be informative for other transcription factors that are sensitive to DNA methylation. CREB has a CpG dinucleotide in its binding sequence and its binding is sensitive to DNA methylation. We applied our method to the analysis of CREB sites and compared our predictions with a previous ChIP–chip study [26] of 10,209 distinct promoters on the human genome that were predicted to have at least one cAMP-responsive element defined as a match to a simple cAMP-responsive element consensus-site algorithm. Forty of these sites were confirmed by manual ChIP assays. Applying our model to these 40 genes, we correctly classified 21 of the 23 high CREB occupancy genes with seven false positives (Table 4). The ChIP–chip assay identified 2,195 CREB high-occupancy binding sites near promoters. Our model correctly classified 1,713 (78%) of these high-occupancy promoters and gave 3,621 false positives (Table 5). However, many false positives could actually be true positives, because Zhang et al. pointed out in their paper that, although their ChIP–chip method had a high specificity, only 54% of the promoters occupied by CREB in their manual ChIP assay showed positive in their ChIP–chip assay. These predictions on CREB show that our method is useful for the analysis of other transcription factors that are sensitive to epigenetic factors.

**Table 4:**
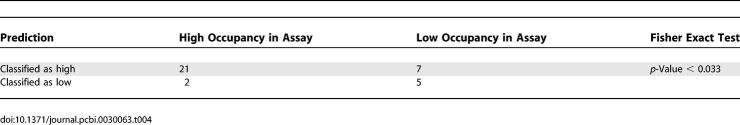
Prediction of CREB Occupancy in Genes Manually Assayed by ChIP

**Table 5:**
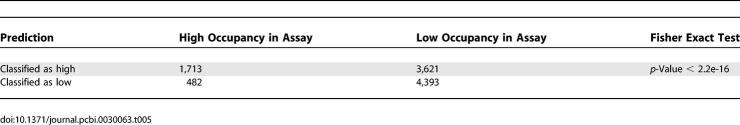
Prediction of CREB Occupancy in Promoters Assayed by ChIP–chip

### Gene Expression Analysis

Myc binding does not always imply c-*MYC* regulation, and a great deal remains to be learned about how the cell determines which genes are actually regulated by c-*MYC* under different conditions and in different tissues. To address this issue, we analyzed the co-expression pattern of genomic genes and c-*MYC* genes in two tissues where c-*MYC* is reported to play an important biological role: B cell lymphoma and prostate cancer.

**Figure 3:**
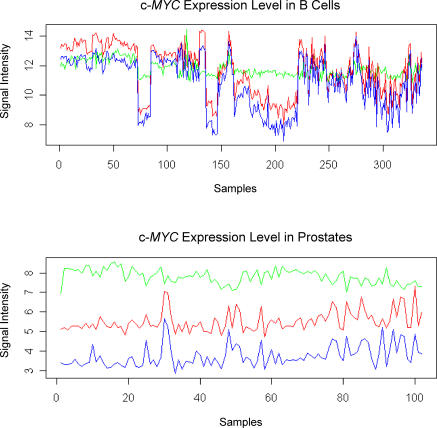
c-*MYC* Expression Level in Both Tissues The RMA normalized signal intensity of c-*MYC* probe sets in B cells (upper) and prostates (lower). Green, c-*MYC* probe set “1827_s_at”; red, c-*MYC* probe set “1973_s_at”; blue, c-*MYC* probe set “37724_at.”

**Figure 4:**
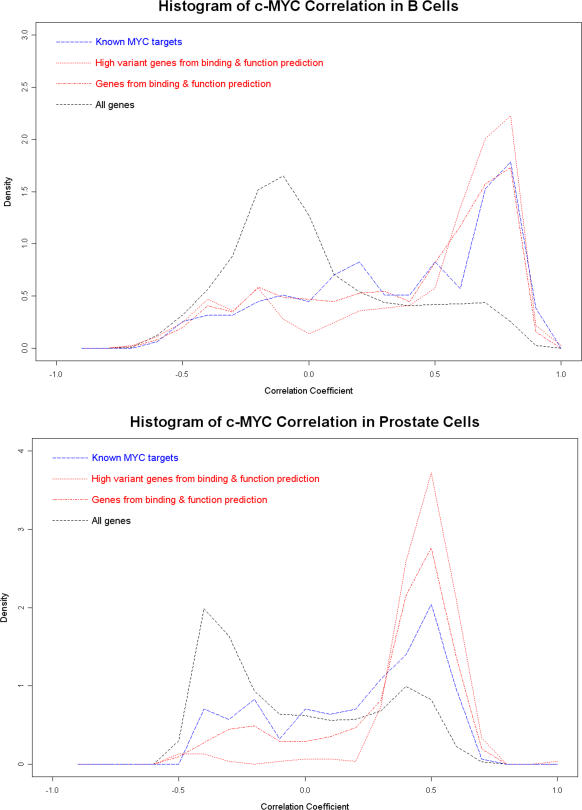
Distribution of c-*MYC* Co-Expression Coefficients in B cells and in Prostate Cancers Distribution of correlation coefficients for c-*MYC* with genes in B cells (A) and prostate cancers (B). The predicted genes in Figure 4 refer to the high-occupancy MYC binding and MYC target gene predictions.

The c-*MYC* gene is often deregulated in cancer and induces the expression of many c-*MYC* target genes. Notable examples include B cell lymphoma and prostate cancers. We selected two datasets for analysis, a human B cell dataset [14] of 336 samples and a human prostate cancer dataset [37] of 102 samples based on number of samples in the dataset, availability of the raw data, and thus use of a standard gene expression analysis platform, the Affymetrix HG-U95Av2 microarray. The raw data for both datasets were reprocessed with Bioconductor [38] and the RMA algorithm [39]. Figure 3 shows the RMA normalized log transformed expression signals of the three c-*myc* probe sets on the HG-U95Av2 GeneChip for each dataset. In this study we used two c-*myc* probe sets, “1973_s_at” and “37724_at”, because their expression signals demonstrated a strong and consistent correlation, whereas “1827_s_at” was not strongly correlated with either of the other two (see Figure 3). The Pearson's correlation coefficients between each c-*myc* probe set and every other probe set on the chip was calculated, and this value is referred to as the co-expression pattern of c-*myc* with the other genes. Figure 4 shows the distribution of these co-expression patterns for data derived from B cells and prostate cancers. Overall, 1,217 and 1,418 genes were found to be significantly correlated with c-*MYC* expression (FDR < 0.01) in B cells and prostate cells, respectively. Altogether, 2,233 genes were highly correlated with c-*MYC* in these two datasets; 403 genes were correlated with c-*MYC* expression in both datasets. Table S1 lists the MYC correlated genes identified in these two tissues.

### Gene Function Annotation

Many known MYC target genes have functions involved in cell cycle progression, apoptosis, and cellular transformation; it is reasonable to hypothesize that unknown MYC targets will share many of these functions. We constructed a set of gene functions that were overrepresented in known MYC targets as represented in GO terms. Using the experimentally verified MYC targets in the MYC target database [17] and considering only the genes on the HG-U95Av2 chip, we found 144 known MYC targets to which 875 terms in the *Molecular Function* and *Biological Process* categories were applied. Because some GO terms are too general to be as informative, in subsequent analysis we only used the GO terms with fewer than 500 genes. Applying a hypergeometric test, we found 156 GO terms overrepresented in the MYC target gene sets (*p*-value < 0.025; Table S2). Among the top overrepresented GO terms are those related to cell cycle, biosynthesis, nucleic acid binding, and translational regulation. These findings correlate well with expectations based on the biology of c-*MYC*.

### Integrated Approach for Predicting MYC Target Genes

Using the predicted MYC high-occupancy binding sites, the c-*MYC* co-expressed genes in B cells and prostate cells, and genes annotated with GO terms that are overrepresented in known c-*MYC* targets, we applied a rule-based procedure to define likely c-*MYC* targets. For a gene to be labeled as a c-*MYC* target, it must meet four criteria. First, the gene must have at least one MYC high-occupancy binding site. Second, the signal variance of the gene's probe set must be greater than the mean variance across all probe sets in either the B cell or prostate cancer dataset. Third, the correlation coefficient must be greater than zero and the significance of gene's co-expression with c-*MYC* must be less than 0.01 in either the B cell or prostate cancer dataset. Fourth, the GO annotation for this gene must have at least one overrepresented c-*MYC* target-related GO term.

Applying these rules, we found 440 genes that meet our criteria (see Table S3). Comparing our findings with the Myc Target Gene Database [17], we found that in the literature, 128 of the predicted target genes are reported to bind MYC and 142 are reported to be regulated by MYC. This represents an independent validation of our findings because data from the MYC Target Gene Database was not included in our training data. Sixty-two of the predicted target genes were reported to be both bound and regulated by MYC; we successfully predicted 62 out of the 144 known MYC targets on HG-U95Av2 platform.

**Figure 5:**
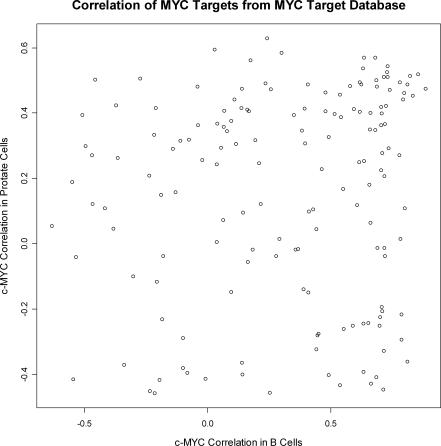
Expression of Known c-*MYC* Targets in Both Tissues Pearson's correlation coefficient of MYC target genes in B cells and prostate cancers. The MYC target genes were from MYC Target Gene Database.

Among the predicted genes, 264 were correlated with MYC expression in B cells, 277 were correlated with MYC expression in prostate cancers, and 101 were correlated in both datasets. This high level of correlation further validates our predictions. Among the 62 previously identified targets, 42 correlate with MYC expression in the B cell dataset, 39 in the prostate dataset, and 19 in both. These findings show that many MYC targets exhibit some tissue specificity in MYC responsiveness. Figure 5 also shows that many of the known MYC targets in the MYC Target Gene Database [17] have different correlations of gene expression with MYC expression in the two tissues.

To further investigate c-*MYC* tissue specific responses, we examined seven additional microarray gene expression datasets from breast, lung, prostate, and leukemia cancers (Table 6). All these datasets used the Affymetrix HG-U95Av2 platform. Adding correlation of gene expression with c-*MYC* in these datasets, we were able to identify 20 additional MYC targets. In total, 460 c-*MYC* target genes were predicted including 215 in the MYC Target Gene Database that had previously been reported to be bound or regulated by MYC (Table S3); 144 were regulated by MYC and 132 were bound by MYC. Further, we found evidence in the literature to validate three additional MYC target gene predictions, ATF3 [40], HSP90A [41], and BAT1 [42]. Overall, 218 of our 460 predictions were validated, including 67 genes that have evidences for both binding and regulation. We believe that this is an underestimate for the true number of MYC targets because we only considered the 8,000 GO annotated genes on Affymetrix HG_U95Av2 platform; this is slightly more than one-third of the genes in the human genome.

**Table 6:**
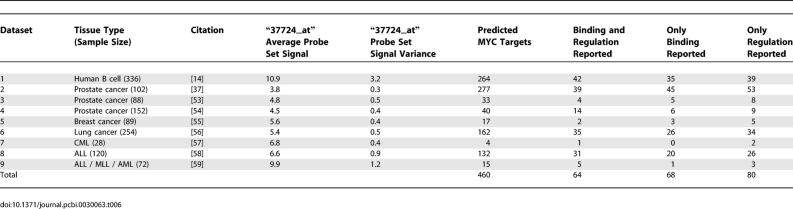
Summary of MYC Targets Prediction from Different Microarray Datasets

**Figure 6:**
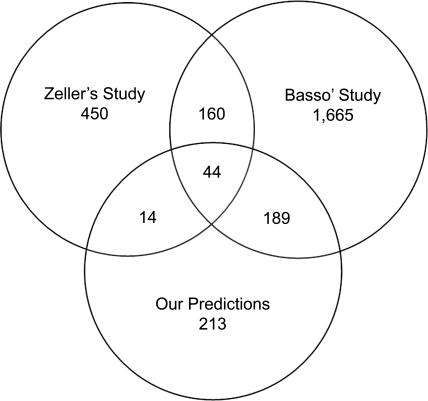
Overlaps of c-*MYC* Targets Genes Venn diagrams for the overlap between the set of c-*MYC* target genes identified in the different experimental and theoretical studies.

We compared our predicted 460 genes from nine datasets with the 2,063 genes in the MYC subnetwork predicted by Basso et al. [14] and the 668 high-quality MYC direct responsive genes identified by Zeller et al. [35]. Figure 6 shows the overlaps between these datasets. We see that the overlap number is higher than would be expected purely by chance, but well below complete agreement. Comparing these datasets with the genes in the MYC Target Gene Database (Table 7), we find that our method shows a better specificity than the other two approaches, even without applying GO filtering. Using the GO functional annotation improved the specificity. We investigated the 45 targets identified by Basso et al. that were missed in our 460 predicted targets. Experimentally, these targets exhibit both binding and regulation. Thirteen targets were missed due to their relatively low expression variation or nonpositive correlation with c-*MYC* across samples, six targets did not have any c-*MYC* binding motif within 3 Kb of their transcription start sites, 15 targets were false negatives in high-occupancy sites prediction, and the other 11 targets did not have overrepresented GO terms.

**Table 7:**
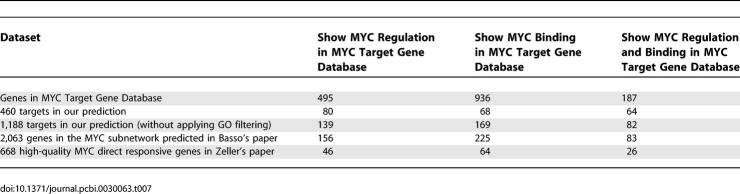
Comparison of Different MYC Binding Datasets to Genes in MYC Target Database

## Discussion

Using a Bayesian model, we integrate genome sequence data and epigenetic information to identify myc recognition sites in the human genome likely to bind c-*MYC* with high occupancy. By combining the myc binding probability, gene co-expression data, and functional annotations, we predicted 460 c-*MYC* targets among the genes presented on Affymetrix HG-U95Av2 platform. The list of predicted c-*MYC* targets contains many genes found previously in the literature, but also 245 genes not previously identified as c-*MYC* targets. Our method only predicts upregulated c-*MYC* targets because downregulated c-*MYC* targets are not generally mediated by E-box binding. Among the 67 predicted genes that have already been observed to be bound and regulated by MYC, 61 are upregulated by MYC, five genes are reported to be downregulated, and one has evidence for both downregulation and upregulation. Thus, the predicted MYC targets agree well with previous observations. In addition to these 67 independently validated predictions, we also identify 148 genes in the MYC Target Database, 68 of which are bound by MYC in vivo and 80 of which are MYC regulated (Table 6). Among the 250 predicted novel targets, 27 correspond to MYC binding loci reported by Zeller et al. [35] and 11 of these correspond to highly reliable binding loci with PET-3+ clusters.

Different from previous studies predicting c-*myc* targets, our study integrated four sources of data (genomic sequence, gene expression, ChIP, and functional annotation) to improve the specificity of predictions. Tools such as TRANSFAC or MatInspector, which rely on motif matches alone to predict myc binding, have very high false positive rates. The improved specificity obtained by our integrated approach emphasizes the importance of epigenetic factors in modulating c-*MYC* binding to DNA. Although epigenetic status does vary with tissue type and other factors, recent high throughput studies show that tissue-specific variation in genomic methylation is limited [27]. Adding genomic acetylation islands data obtained on T cells to the model improved the prediction precision from 0.81 to 0.88 and the recall from 0.88 to 0.98 in cross-validation. Among the attributes considered in the model, we found that the distance to the nearest hypomethylation region is the most informative; this attribute alone could correctly identify 80% of the high- and low-occupancy sites in the cross validation. However, adding the additional attributes does improve performance, and considering all five attributes allows the model to correctly identify 95% of all the cases in cross-validation.

MYC is not the only transcriptional factor whose binding is sensitive to the epigenetic factors such as DNA methylation or chromatin acetylation. Because the attributes used in our model are general and the epigenetic factors could influence DNA binding through similar mechanisms, elements of our model may also be useful for other transcription factors. As a validation we applied our MYC binding prediction model to another transcription factor, CREB [26]. Like MYC, the CREB consensus binding sequence has a CpG dinucleotide and CREB binding is sensitive to DNA methylation. Our analysis shows that although our model was specifically built for the study of c-*MYC*, it is still able to correctly discriminate most of the high-occupancy binding sites and the majority of low-occupancy binding sites. Thus, our approach to modeling chromatin structure effects is transferable to other transcription factors. ChIP–chip technology can partially address the question of where transcription factors bind the genome, but with current technology the resolution of TF binding loci is limited and the data are error-prone. In addition, computational modeling can help to understand the complex transcriptional machinery. For example, adding or removing an attribute to the model and assessing the affects on performance is one way to evaluate the importance of this attribute on the regulation of the DNA binding by a transcription factor.

Combining different types of data offsets the shortcoming of each. Obviously, our predictions based on genome sequence alone cannot address tissue specific binding. However, by taking gene expression data of specific tissues into consideration, we restrict our identified targets to those functionally regulated by MYC in the context of certain tissues, which would be of real interest to the biological community. In addition, integration of different types of data also allows us to predict which DNA–protein binding sites are likely to trigger transcriptional regulation. Figure 4 shows that compared with the expression of all genes, a higher portion of genes from our MYC binding and function predictions are highly correlated with c-*MYC* expression. This demonstrates that our binding prediction and gene function analysis do identify bona fide c-*MYC* targets and are helpful in improving the specificity of prediction.

One of the limitations in our target gene analysis is that we only consider genomic sequences within 3 Kb of transcription start sites. This was done because almost all of the sites in our training data fall within this region, but there are some high-occupancy MYC target sites far from any known transcription start sites. It is possible that there are direct c-*MYC* targets where the only functional MYC sites are more than 3 Kb from the transcription start site. Increasing the search window for c-*MYC* recognition sites might improve the sensitivity of prediction, but such a change would also decrease specificity.

Another limitation of our method is that we only consider E-box–dependent MYC binding and regulation; MYC targets regulated through other mechanisms will not be identified. Transcriptional inhibition of MYC targets is often mediated by mechanisms unrelated to E-box binding [43–47]. Therefore, we only consider the MYC activation in our predictions and consider only the expression of target genes that is positively correlated with c-*MYC* expression. We do observe a minority of genes where there is a significant anti-correlation of gene expression with c-*MYC* expression. The analysis of these cases will be the subject of future work.

Distinguishing between direct and indirect targets of MYC is an important issue. In the co-expression analysis we found a large number of genes where expression levels showed positive correlations with c-*MYC* expression and yet lacked a high-occupancy MYC binding site, either predicted or experimental. We believe that many of the genes are not the direct targets of MYC, but it is difficult to exclude the possibility that they contain a functional MYC binding site not detected by our own or experimental methods.

MYC responses vary significantly between different tissues. Less than half of our predicted MYC targets show significant gene expression correlation with c-*MYC* in both B cells and prostate cancers. We analyzed seven additional independent microarray datasets (Table 6) where the c-*MYC* gene was deregulated and its probe-set signals showed large variance across samples. In these studies, many predicted MYC targets, including genes that have been experimentally verified as direct MYC targets, failed to show a strong correlation of target gene expression with MYC expression. The differences between these datasets cannot be explained by tissue of origin alone. For example, datasets 2, 3, and 4 are all derived from the prostate, but the c-*MYC* correlated targets from these three datasets do not agree more than those from different tissues (see Figures S3–S5). Some of this variation could be a result of technical variation in gene expression profiles between different laboratories and experiments.

This is one of the first studies to systematically analyze c-*myc* targets in multiple datasets and in multiple tissues; most previous studies focused on a single tissue or cell line. Our analysis confirms the well-known finding that c-*MYC* is deregulated in many cancers and has a direct influence on the expression of hundreds of other genes. One potential pitfall in using GO as a criterion for predicting MYC targets is the possibility of missing important groups of targets that do not fall into a specific GO category or are not annotated by GO at all. In Table S3 we also provide the list of 1,188 predicted targets without applying GO filtering in the prediction. A second concern is that it is difficult to define a test set that is totally independent of prior knowledge because we cannot exclude the possibility that GO annotators were aware of MYC regulation status in assigning gene annotations.

Looking at the target genes we predicted that were not in the Basso et al. prediction, we find variable levels of correlation with MYC expression across the different datasets that we have examined. Even genes that have been identified as c-*MYC* targets in published literature often have very different co-expression patterns with c-*MYC* in different microarray datasets. This is consistent with the view that many of the differences among these high throughput studies may result from experimental variation, the noise inherent in these approaches, and the effects of cell density or the number of culture passages [48]. Whether this reflects tissue-specific responses or technical variation in microarray data, it is apparent that a study focusing on any single dataset will be insufficient. As is shown in Table 6, using multiple datasets from different studies improved the power of the prediction.

Because the current study only predicted upregulated genes with MYC binding motifs close to the transcription start site, which are on the Affymetrix HG-U95Av2 array and which have GO annotations, we believe that the 460 targets identified here underestimate the number of direct MYC targets in the human genome. Our estimates for the number of MYC targets in the human genome are roughly consistent with the MYC database and the high-confidence Zeller [35] and Cawley [36] studies. They are not inconsistent with the larger numbers of c-*MYC* targets suggested by some other studies [12–15]. One explanation for this range of findings is that MYC binding in vivo is not a Boolean event and even strong MYC binding sites are unlikely to be occupied with unit stoichiometry. Thus, different studies may be applying different thresholds for defining a MYC target.

## Materials and Methods

### Data sources.

All the genes in the paper and their Entrez Gene IDs can be found in Tables S1 and S3.

The HG17 build of the human genome sequence was downloaded from the University of California Santa Cruz (UCSC) genome database. The transcription start sites were from the annotated transcription starts of RefSeq genes in the UCSC genome database (http://hgdownload.cse.ucsc.edu/goldenPath/hg17/bigZips/upstream1000.zip). PhastCons scores for multiple alignments of seven assemblies to the human genome hg17 were downloaded from http://hgdownload.cse.ucsc.edu/goldenPath/hg17/phastCons/mzPt1Mm5Rn3Cf1Gg2Fr1Dr1/. The human gene annotation information was downloaded from ftp://ftp.ncbi.nlm.nih.gov/gene/DATA/gene2refseq.gz, and the GO information was downloaded from ftp://ftp.ncbi.nlm.nih.gov/gene/DATA/gene2go.gz.

Known MYC target genes in previous literature were obtained from the MYC Target Gene Database [17] (http://www.myc-cancer-gene.org). The large-scale assay for genomic MYC binding sites in live human cells was obtained from the supplementary data of Fernandez et al. [19]. Chromatin acetylation data were obtained from the supplementary data of Roh et al. [33]. MYC binding loci on Chromosomes 21 and 22 [36] were downloaded from http://transcriptome.affymetrix.com/publication/tfbs/, and MYC binding data in human B cells using ChIP–PET were obtained from the supplementary data of Zeller et al. [35]. The CREB genomic binding loci [26] was downloaded from http://natural.salk.edu/CREB. Two microarray gene expression datasets were used in this study, the B cell dataset [14] (GSE2350) and the prostate cancer dataset [37] (http://www.broad.mit.edu/cgi-bin/cancer/datasets.cgi). The B cell dataset contains 336 samples of normal and transformed human B cells, and the prostate dataset contains 52 tumor and 50 nontumor prostate samples. The sources of other microarray gene expression profile datasets are listed in Table 6.

### Construction of the Bayesian network.

Bayesian network classification was performed using *Weka 3.4.5* [34], which is available for download at http://www.cs.waikato.ac.nz/ml/weka/. When learning the Bayesian network, the best performance was obtained when using *K2* as the search algorithm for local score metrics and using *Simple Estimator* with *alpha* at 0.5 to find the conditional probability tables. In addition, an empty network is set as the initial structure when learning the network.

The training data contained 43 high-occupancy sites and 90 low-occupancy sites from Fernandez's dataset [19]. These sites and their ChIP primers were mapped back to human genome using the *UCSC In-Silico PCR* tool. The five attributes for every site were derived from the genome sequence and annotations, including: distance to transcription start site, distance to nearest CpG island, distance to nearest hypomethylation region, nearest hypomethylation region score, and phastCons scores [32]. The CpG islands and hypomethylation status of sequence were predicted as described below. The distances in base pairs and the hypomethylation scores were transformed to natural log scale.

### Prediction of CpG islands and hypomethylation regions.

CpG islands were predicted by the criteria of Gardiner-Garden [49]. Hypomethylation regions were predicted using a fifth-order Markov model likelihood ratio test.

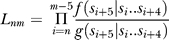
where *L_nm_* is the likelihood ratio score that the hexamers in the interval from *n* to *m* are drawn from the frequency distribution for a training set of hypomethylated sequences relative to the likelihood that they are drawn from a frequency distribution describing the genome as a whole. *s_i_* is the residue occurring at position *i* in the sequence, *f*(*s_i_*_+5_ | *s_i_*…*s_i_*_+4_) is the frequency of finding residue *s_i_*_+5_ given the preceding five residues *s_i_*…*s_i_*_+4_ in the hypomethylated DNA collection, and *g*(*s_i_*_+5_ | *s_i_*…*s_i_*_+4_) is the corresponding frequency in the genome as a whole. The hypomethylation score for a region is the sum of the log likelihood ratio scores for all overlapping hexamers in the region, expressed as log base 2. This score measures how similar the hexamer content of a region is to the hexamer frequencies in the hypomethylated sequences training set described below. The boundaries for a predicted hypomethylation region are chosen to maximize this log likelihood ratio score, with constraints that each predicted region must score at least 23.1 bits and must not contain any subregion whose score is more negative than −14.5 bits. An optimized hexamer score cutoff is determined as shown in Figure S1.

This algorithm is implemented in the program FMA, a C++ program, which utilizes a hexamer model to predict hypomethylation regions. The output includes the exact locations of CpG islands, the fraction of CpG dinucleotides, and the exact location and score (log_2_ *L_nm_*) of each hypomethylation region. FMA also implements an efficient indexed motif search. In the first phase of the FMA search, each PSWM in the query set is analyzed and the most informative contiguous six-nucleotide core segment is identified. Next, a branch-and-bound strategy is used to enumerate all hexamers matching this core segment and able to be extended over the full PSWM to achieve a specified log likelihood threshold for the full match. Hexamers in this set are stored in a suffix tree. During the search, the target sequence is first scanned using the suffix tree to find core segment matches. These matches are then extended over the full PSWM to see if they achieve the specified log likelihood threshold. Using this approach, we are able to exhaustively search large target sequence libraries for matches to large query sets of PSWM.

The training data for hypomethylated human genomic sequences were obtained by aligning the hypomethylated sequence tags collected by Cross et al. [50,51] with the human genome sequence and extending to the nearest MseI site. These hypomethylated sequence tag sequences were obtained by digesting human genomic DNA from peripheral blood leukocytes with MseI, selecting fragments that failed to bind a methyl–CpG binding protein column, methylating these fragments in vitro, and subsequently selecting fragments that bound the methyl–CpG binding protein column. This yields a collection of genomic DNA fragments that were not methylated in vivo, but which contained a CpG dinucleotide that could be methylated in vitro. These fragments were subsequently cloned and subjected to end-sequence analysis. By aligning the end-sequence tag to genomic sequence and extending to the nearest MseI site, we reconstruct the sequence of the full hypomethylated DNA segment.

Validation data for hypomethylation predictions were obtained from the Human EpiGenome Project [27].

### Genome-wide prediction of high-occupancy MYC binding sites.

TRANSFAC was used to scan the human genome sequence for putative c-*MYC* binding sites with the MYC–MAX position weight matrix [MA0059] [29] from the JASPAR database [30].

The sequence attributes for each putative binding site were inputted into the Bayesian network classifier (see above) to predict the probability of MYC binding for each site. A site assigned a MYC binding probability above 0.5 was considered to be a high-occupancy site. Because almost all the sites in the training data were within ±3 Kb of transcription start sites, we limited the prediction of high-occupancy binding sites to this region for further studies.

The prediction of c-*MYC* binding genes was based on the high-occupancy c-*MYC* binding sites prediction. If a gene contained any high-occupancy binding site, it was considered a potential c-*MYC* binding gene.

### Gene expression analysis.

The HG-U95Av2 platform annotation file was downloaded from Affymetrix. The raw data files for each dataset were firstly normalized with *RMA* in *Bioconductor* [38]. For each probe set, the signal variance across the samples was calculated. Only probe sets with a signal variance larger than the mean of all probe sets' variances were used in co-expression analysis. The Pearson's correlation coefficient *r* was then calculated for every pair consisting of a c-*MYC* probe set and another probe set. The significance (probability) of the correlation coefficient is determined using the *t*-statistic:

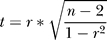
where *r* is the correlation coefficient and *n* is the sample size. The *p*-values from this multiple testing were adjusted to control the false discovery rate of Benjamini and Hochberg [52]. Only probe set pairs with an adjusted *p*-value less than 0.01 were considered to be significantly co-expressed. For a gene to be correlated with c-*MYC* expression, it must have at least one probe set with *p*-value less than 0.01 for both c-*MYC* probe sets “1973_s_at” and “37724_at.”

### Gene function analysis.

We analyzed genes on the HG-U95Av2 GeneChip. From the Myc Target Gene Database [17], we extracted the subset of these genes that were reported to be both bound and regulated by myc. For these genes, we collected the associated GO terms from the *Molecular Function* and *Biological Process* trees and tested for significant overrepresentation using a hypergeometric test implemented in the GOHyperG function Bioconductor [38] package GOstats. A GO term is claimed to be significant if the *p*-value is less than 0.025.

## Supporting Information

Figure S1 Determine the Optimized Hexamer Score Cutoff in the Hypomethylation Prediction Algorithm (63 KB DOC)

Figure S2 Bayesian Classifier Model (3 KB DOC)

Figure S3 Predicted c-*MYC* Target Genes That Are Overlapped in Three Prostate Datasets (98 KB DOC)

Figure S4 Comparison of Correlation Pattern of Reported c-*MYC* Target Genes in Same Tissues (166 KB DOC)

Figure S5 Comparison of Correlation Pattern of Reported c-*MYC* Target Genes in Different Tissues (146 KB DOC)

Table S1 Genes Whose Expressions Are Significantly Correlated With c-*MYC* Expression in Dataset 1 and Dataset 2 (212 KB XLS)

Table S2 GO Terms Overrepresented in Known c-*MYC* Targets (35 KB XLS)

Table S3 Predicted MYC Targets (232 KB XLS)

## Abbreviations

ChIP: chromatin immunoprecipitation
CREB: cAMP-response element-binding protein
FMA: Fast Motif Analyzer
GO: Gene Ontology
PSWM: position specific weight matrix
UCSC: University of California Santa Cruz
