# Supplementary material for: Integration of Genome and Chromatin Structure with Gene Expression Profiles To Predict c-MYC Recognition Site Binding and Function
Source: PLoS Comput Biol. 2007 Apr 6;3(4):e63. doi: 10.1371/journal.pcbi.0030063 (PMC1847699; doi:10.1371/journal.pcbi.0030063)
Supplement: Supplementary file 1 [file pcbi.0030063.sg001.doc]

Figure S1. Determine the optimized hexamer score cutoff in the hypomethylation prediction algorithm

In the hypomethylation prediction model, we determine the optimized hexamer score cutoff by evaluating what percentages of unmethylated and hypermethylated sites in Human Epigenome Project are in our predicted hypomethylation regions. A plot was made in which the Y axis represents the percentage of the HEP unmethylated sites located within the predicted hypomethylated regions and the X axis represents the percentage of the HEP hypermethylated sites located outside our predicted hypomethylated regions.  The points on the curve are the outcomes predicted by the model with different hexamer score cutoffs.  The ideal situation would be a hexamer score cutoff making a point with both a large X-value and a large Y-value.  From the curve, it is not difficult to see that the point with a hexamer score cutoff of 28 maximizes the performance of the model.

The methylated data are from Human Epigenomics Project (Eckhardt F, *Nature Genetics* 2006). The unmethylated sites are those with an average methylation score < 20 and the hypermethylated sites are those with an average methylation score > 80.
