# Supplementary material for: Integration of Genome and Chromatin Structure with Gene Expression Profiles To Predict c-MYC Recognition Site Binding and Function
Source: PLoS Comput Biol. 2007 Apr 6;3(4):e63. doi: 10.1371/journal.pcbi.0030063 (PMC1847699; doi:10.1371/journal.pcbi.0030063)
Supplement: Supplementary file 3 [file pcbi.0030063.sg003.doc]

Figure S3

Figure S3 shows how many predicted c-*MYC* target genes are overlapped in three prostate datasets.
