# Supplementary material for: Integration of Genome and Chromatin Structure with Gene Expression Profiles To Predict c-MYC Recognition Site Binding and Function
Source: PLoS Comput Biol. 2007 Apr 6;3(4):e63. doi: 10.1371/journal.pcbi.0030063 (PMC1847699; doi:10.1371/journal.pcbi.0030063)
Supplement: Supplementary file 4 [file pcbi.0030063.sg004.doc]

Figure S4

Figure S4 shows the comparison of correlation pattern of reported c-*MYC* target genes in same tissues. (X-axis: Pearson’s correlation coefficient from dataset A; Y-axis: Pearson’s correlation coefficient from dataset B)
